# 

**Published:** 1832-10-01

**Authors:** 


### BIBLIOGRAPHICAL RECORD;

OR,

*Works received for Review since the last Quarter.*

1. The Sources of Health and Disease in Communities; or Elementary Views of Hygiene: illustrating its importance to Legislators, Heads of Families, &c. By Henry Belinage, Esq. Small 8vo, pp. 262. Truttel and Wurtz, July, 1832.

2. Two Lectures on the Primary and Secondary Treatment of Burns. By Henry Earle, F.R.S. Surgeon Extraordinary to the King, &c. Octavo, pp. 59. July, 1832.

3. Counteraction, viewed as a Means of Cure; with Remarks on the Uses of the Issue. By John Epps, M.D. Lecturer on Materia Medica, &c. Octavo, pp. 69. July, 1832. ☞ *See Periscope.*

4. Practical Essays on Medical Education in the United States. By Daniel Drake, M.D. Octavo, pp. 104. 1832.

5. A Conspectus of the Butterflies and Moths found in Britain; with their English and systematic Names, Times of Appearance, Sizes, Colours, &c. By James Rennie, M.A. Professor of Zoology, King's College, &c. Duodecimo, pp. 287. July, 1832. Price 7s. 6d. boards.

6. Scientific Alphabet of Insects, for the Use of Beginners. By James Rennie, M.A. Professor of Zoology, King's College, London. Duodecimo, pp. 108. July, 1832.

*The two little works above recorded, comprise, within small dimensions, a vast mass of information. They will be exceedingly useful in acquiring an insight of that very curious and beautiful study,* Insectology. *We strongly recommend these pocket volumes.*

7. Transactions of the Medical and Physical Society of Calcutta. Volume the Fifth. Octavo, pp. 449. Calcutta, 1831.

☞ *Noticed in various parts of this Journal.*

8. Medical Reports: containing Observations on Cholera, Dysentery, the Effects of Sulphate of Quinine as a Remedy of Fever, and on Inflammation of Veins, with Cases of these Diseases, and Notices relative to the Treatment of Syphilis without Mercury, &c. Selected by the Medical Board (Madras) from the Records of their Office, and published under the sanction of Government. 8vo, pp. 363. Madras, 1831. (Presented, with the sanction of Government, to Dr. James Johnson, of London, by the Madras Medical Board.)

☞ *We have given some account of this important volume in this number.*

9. Practical Observations on Midwifery; with a Selection of Cases. Part II. By John Ramsbotham, M.D. late Lecturer on Midwifery at the London Hospital, &c. Octavo, pp. 507. Highley, London. July, 1832.

10. Effects of Arts, Trades, and Professions, and of Civic States and Habits of Living, on Health and Longevity: with Suggestions for the removal of many of the Agents which produce disease, and shorten the Duration of Life. By C. Turner Thackrah, Esq. Second Edit. greatly enlarged. July, 1832.

11. Moyens à opposer au Cholera Pestilentiel; fautes qu'on doit eviter. Par J. N. Guilbert, ancien Professeur de la Faculté de Medicine de Paris. July, 1832.

12. Notes and Observations upon the Contagion of Typhus Fever, and Contagion generally. By William Fergusson, M.D. F.R.S.E. &c. (Corrected from the Edinb. Med. and Surg. Journal.)

13. Clinical Reports of the Surgical Practice of the Glasgow Royal Infirmary. By John Macfarlane, M.D. Octavo, pp. 314. Glasgow, July, 1832.

14. Statement of Facts with Observations. By Dr. Craigie, of Leith. 8vo, sewed. July, 1832.

15. Observations on Spasmodic Cholera, its Origin, Nature, and Treatment; with Remarks on Epidemic Diseases generally. By Henry M'Cormac, M.D. Belfast, July, 1832. 8vo, sewed, pp. 20.

16. Tabula Anatomiam Comparativam Illustrante, quas exhibuit Carolus Gustavus Carus, M.D. &c. Textum in Latinum Sermonen Vertit F.A.L. Threne-

MANN, M.D. Pars III. Continens Novem Tabulas, æri incisas, variarum Animalium Classium Historiam Evolutionam Illustrantes. Folio. Lipsiæ, 1831.

☞ *Noticed in the Journal.*

---

17. A plain and brief Sketch of Cholera, with a simple and econominal Mode for its Treatment, submitted with confidence from repeated success in its Application. By WILLIAM HENRY WILLIAMS, M.D. F.R.S. &c. Second Edition, revised and enlarged. Octavo, stitched, pp. 29. Ipswich, 1832.

☞ *Noticed in Periscope.*

---

18. A Compendium of Human and Comparative Pathological Anatomy. By ADOLPHO WILHELM OTTO, M.D. Royal Counsellor in the Medical College of Silesia, &c. Translated from the German, with additional Notes and References. By JOHN F. SOUTH, Lecturer on Anatomy at St. Thomas's Hospital. 8vo, pp. 456. London, 1831. ☞ *Noticed in this No.*

---

19. The Anatomy and Physiology of the Organ of Hearing; with Remarks on Congenital Deafness, the Diseases of the Ear, some Imperfection of the Organ of Speech, and the proper Treatment of these several Affections. By DAVID TOD, M.R.C.S. 8vo, pp. 147. London, 1832.

---

20. Dr. Davis's Principles and Practice of Obstetric Medicine. Part X.

☞ *To be noticed.*

---

21. The Cyclopædia of Practical Medicine for June, July, August.

☞ *To be noticed.*

---

22. Observations on Spasmodic Cholera, its Origin, Nature, and Treatment; with Remarks on Epidemic Diseases generally. Second Edition. 8vo, stitched, pp. 28. London, 1832.

☞ *A sensible pamphlet.*

---

23. A Treatise on Cholera, containing the Author's Experience of the Epidemic known by that name, as it prevailed in the City of Moscow in Autumn, 1830, and Winter, 1831. By JAMES KEIR, M.D. &c. Octavo, pp. 138. Edinburgh, 1832.

24. Experimental Inquiries in Chemical Physiology. Part I, complete in one volume, on the Blood. With an Appendix, containing Remarks on the Nature and Treatment of Cholera Asphyxia. By HORATIO PRATER. 8vo, pp. 304. Highley, London, 1832.

25. De la Nature, du Siege, et du Traitement du Cholera-Morbus. Par M. M. FOVILLE et PARCHAPPE, Docteurs en Médecine. En 8vo, pp. 64, 2 planches. Rouen, 1832.

☞ *We shall notice this pamphlet.*

---

26. A Letter addressed to the Central Board of Health, written with the View of establishing rational Principles for the Treatment of Cholera; and shewing the Danger of the Mode of Practice at present generally followed. By JOHN GEO. FRENCH, Surgeon to St. James's Cholera Hospital, &c. Octavo, stitched, pp. 22. London, 1832.

---

27. The Principles and Practice of Obstetric Medicine. By Dr. D. DAVIS, &c. Part XI.

---

28. Denman's Introduction to the Practice of Midwifery. Seventh Edit. With additional modern Information on the various Subjects treated of in the Work, and a Dissertation in the Transfusion of Blood in the more dangerous Varieties of Uterine Hæmorrhage. By CHAS. WALLER, M.D. &c. 8vo, pp. 525. Cox, London, 1832.

---

29. Surgical Anatomy of the Arteries. With Plates and Illustrations. By NATHAN R. SMITH, M.D. Professor of Surgery in the University of Maryland, and one of the Surgeons of the Baltimore Infirmary. 4to, bds, pp. 104, 18 plates. Baltimore, 1832. ☞ *To be noticed.*

---

30. On a New Membrane in the Eye; being the Substance of a Lecture delivered at Oxford before the British Association for the Advancement of Science. By GEO. HEMSLEY FIELDING, M.R.C.S.L. &c. 8vo, stitched, pp. 28. Hull, 1832.

☞ *To be noticed.*

---

31. Parts I. and II. of the Illustrations of the Surrey Zoological Gardens, drawn from Nature on Stone, with descriptive Notices. By W.H. Rearney. Lithograph. Prints, 3s. 6d. each. Proofs on India, 6s.

☞ *These are extremely good lithographic plates, and must prove an acceptable work to the lovers (as who are not?) of natural history.*

*We have also received from Mr. Schloss a coloured lithograph by L. Stole, a very promising artist, representing some of the parrot tribe. It is executed clearly, neatly, and cleverly.*

### INDEX.

A.

Abdomen, case of wound of ....... 226
Abdominal parietes, spontaneous perforation of ................. 441
Abortion, Mr. Ingleby on ........ 342
Abscess of liver, cases of.......... 523
Abscesses, fetid, near mucous membranes ........................ 433
Accidental colours, Sir I. Newton's case.......................... 471
Acid nitrate of mercury, formula of, 368
Acoustic illusions, Sir D. Brewster on.............................. 479
Acoustics on heights and at great depths ........................ 461
Actual cautery, utility of.......... 164
Allsop, Dr. death of ............... 574
Amaurosis, case of ............... 282
Amputation of fingers, fatal, cases of 530
Analysis of the Bombay reports on cholera ......................... 65
Anatomical models, Mr. Schloss's.. 564
Anatomy, pathological, of Otto .... 427
Anatomy, &c. of the ear, Mr. Tod's, 431
Anatomy, comparative, Carus's.... 501
Anatomy Act...................... 566
Aneurism of the brachial artery.... 204
Aneurism, cases of, cured by compression ........................ 204
Aneurism, by anastomosis, case of, 231
Aneurism, flat ligatures in ........ 444
Angina externa, remarks on ...... 416
Angina pectoris—diseased heart .. 463
Animal heat, Dr. Stevens' notions on 325
Anthelmintic emulsion .......... 201
Aorta, pressure on, in uterine hæmorrhage ......................... 358
Apertures of the pelvis, dimensions of.............................. 28
Aphonia treated by argenti nitras.. 435
Apoplexy, M. Dance on a species of, 186
Apoplexy, case of................. 187
Apoplexy of uterus .............. 319
Arnott, Mr. letters to and from.... 503
Arnott's, Dr. hydrostatic bed...... 564
Arsenic, poisoning by ............ 162
Arsenic, Mr. Rainey on .......... 198
Arteries, torsion of the, after operations ........................ 174
Arteritis......................... 146
Arteritis, cases of................ 149
Auscultation, application of to midwifery........................ 163
Auscultation, Dr. Forbes on ...... 246
Auscultation of the heart in the fœtus 246
Auscultation, as a sign of pregnancy 343

B.

Barry, Sir David, and Dr. Stevens.. 422
Barry, Sir David, his visits to Coldbath Prison .................... 423
Bengal reports on the cholera ...... 72
Biett on diseases of the skin ...... 360
Bite from karrait snake .......... 525
Blackmore, Dr. on pulmonary consumption ..................... 400
Bladder, description of .......... 27
Bladder, ruptured, case of ........ 540
Blood-vessels, curious distribution of 178
Blood and blood-letting .......... 249
Blood, Dr. Stevens on the ........ 321
Board of Health, circulars of .. 546 559
Bombay reports on the epidemic cholera ....................... 57
Bones, soup prepared from........ 183
Bones, scrofulous disease of the.... 439
Botany, medical, Mr. Reid's ...... 387
Brazil, medical journal of ........ 182
Breech-presentations, Dr. Ramsbotham on ..................... 289
Breech-presentations, cases of .... 291
Brewster, Sir David, on ocular illusions ....................... 467
Broussais, M. on the chronic phlegmasiæ........................ 158
Bronchitis, Dr. Williams on ...... 250
Burns, Mr. Earle on.............. 492
Burns, opposite plans of treating.. 495
Burns, Mr. Earle's plan .......... 496
Burns, effects of stimulants in .... 497
Burns, secondary effects of........ 498
Burns, contractions after.......... 499

C.

Calculi of large size, Dr. King on .. 40
Capillaries, state of, in inflammation 411
Carbuncular inflammation of fingers 418
Carus' comparative anatomy ...... 501
Case, specimen of a .............. 403
Cataract, cases of................ 282
Catheter slipped into bladder; lithotomy ....................... 528
Cazenave on diseases of skin ...... 360
Cerebellum, deficiency of ........ 177
Cervix femoris, Mr. Green on fracture of the...................... 150

Chimney-sweeper's cancer, Mr. Earle on .......................... 153
Chlorotic diseases, memoir on ..... 179
Chlorotic diseases, cases of......... 180
Cholera, Bombay reports on the .. 57
Cholera, commencement of in Bombay .......................... 59
Cholera, predisposing causes of .... 62
Cholera, treatment of ............ 63
Cholera, analysis of cases of ....... 65
Cholera, abstract of cases ......... 69
Cholera, Bengal report on the .... 72
Cholera, atmospheric changes previous to ...................... 74
Cholera at Jessore ............... 76
Cholera, progress of ............. 76
Cholera, ravages of, in the grand army ........................ 79
Cholera, eccentricity of, in Bengal.. 85
Cholera, symptoms of ............ 88
Cholera, consecutive fever ......... 90
Cholera, remote causes of the ..... 93
Cholera, influence of locality on .. 96
Cholera, contagious nature of the.. 98
Cholera, instances of immunity from 103
Cholera, mortality of, in the army.. 109
Cholera of Newburn, Dr. Craigie on 121
Cholera, propagation of .......... 128
Cholera, Dr. Kirk on the ......... 159
Cholera, Baron Dupuytren's letter on 166
Cholera, Dr. Ochel's letter on .... 171
Cholera, Professor Delpech's letter on the......................... 174
Cholera, account of, in Paris ...... 189
Cholera in Vienna, account of .... 199
Cholera, Broussais' lectures on .... 199
Cholera, Mr. Lizars' letter on....... 229
Cholera, importation of .......... 234
Cholera at Paris ................ 234
Cholera at Ely .................. 235
Cholera, Baron Heurteloup on .... 236
Cholera, modification of opinion on, 237
Cholera in Glasgow .............. 238
Cholera in Cork ................. 239
Cholera, saline injections in ...... 240
Cholera in the ship Brutus......... 285
Cholera in Cold-bath Fields ...... 330
Cholera, Dr. Stevens' treatment of, 331
Cholera again in Cold-bath Fields.. 335
Cholera, Dr. Webster on.......... 383
Cholera, French mandate on ...... 445
Cholera, French official report on.. 448
Cholera, remarks on.............. 544
Cholera, contagiousness of........ 545
Cholera, circular of the Board of Health on ..................... 546
Cholera, moveable commission for.. 547
Cholera—Manchester riot ........ 548
Cholera Excerpta................ 550
Cholera, saline injections into the veins ......................... 550
Cholera, iodine for ............... 550
Cholera, bandaging the abdomen for 551
Cholera, anodyne paste for......... 551
Cholera, cold water for .......... 551
Cholera, narcotic glysters for...... 552
Cholera, prohibition of drink for .. 553
Cholera, mortality from, in London 553
Cholera, Dr. Gregory on.......... 554
Cholera, salines unsuccessful in.... 555
Cholera, Dr. Lindsey's treatment of, 555
Cholera, treatment of, at Leeds.... 556
Cholera, cured by calomel and opium 556
Cholera, a logical deduction ...... 556
Cholera, cold water in............ 556
Cholera, M. M. Foville and Parchappe on ....................... 558
Cholera, last circular on.......... 559
Cholera, editor's remarks on ...... 562
Chronic phlegmasiæ, history of the, 151
Chronic ophthalmia, case of ...... 166
Classifications in botany .......... 395
Clement, Mr. on surgery and pathology.......................... 43
Clinical reports, Mr. Macfarlane's.. 515
Cold-bath Prison, dreadful mortality in ........................... 423
Colours, insensibility to .......... 472
Concussion of the spine, cases of .. 9
Concussion of the spine, consequences of...................... 13
Constipation, fatal case of ........ 530
Consumption, Dr. Blackmore on .. 400
Contagion, Dr. Esquirol on........ 183
Contagion, fundamental doctrine of, 239
Contagion, Dr. Ferguson on ...... 465
Convulsions, puerperal, Dr. Ramsbotham on...................... 304
Convulsions, puerperal, cases of ... 305
Convulsions, cold affusion in ...... 459
Cooper, Sir Astley, on the thymus gland ........................... 139
Cord, diffused hydrocele of the .... 529
Cornea, interstitial abscess of the.. 276
Cornea, ulcer of the.............. 277
Counter-irritation, remarks on .... 415
Crampton, Mr. on injuries of the head ........................... 507
Cranium, on compound fracture of, 511
Cuvier, last illness and memoir of.. 451
Cyclopædia of practical medicine .. 246
Cystocele, fatal case of............ 228

D.

Death of Dr. Allsop .............. 574
Delirium tremens, case of ........ 173
Diabetes, analysis of blood and urine in............................ 219
Diabetes mellitus relieved ........ 439
Dill, Dr. the late ................ 285
Diseases of the spine, Mr. Stafford on 1
Doctrine homoiopathique ........ 132

Dropsy of the membranes ........ 312
Ducamp, Dr. on retention of urine, 49
Dura mater, fungus of the ........ 483
Dysentery, report on ............. 409

E.

Earle, Mr. on chimney-sweeper's cancer ........................ 153
Earle, Mr. on burns............... 492
Electricity, connected with cholera, 163
Elephantiasis Arabum, M. Biett on 379
Elliott, Mr. his case of injury of the head ........................ 538
Emphysema of the lungs, marked case of ....................... 453
Ephelides, M. Biett on ........... 361
Epidemic cholera, Bombay reports on the......................... 57
Epidemic in the northern circars in 1831 ......................... 73
Epidemic cholera, Dr. Webster on.. 383
Epidemic disease among poultry .. 439
Epidemic sweating fever in France, 446
Epidermis of plants .............. 392
Epilepsy, pathology of............ 178
Epilepsy with scrofulous disease of cerebellum...................... 438
Ergot in uterine hæmorrhage ..... 340
Eustachian tube, mode of passing a bougie ....................... 432
Excerpta choleralogica ........... 550
Extra-uterine pregnancy, cases of.. 310

F.

Falrich, Dr. his wax models ...... 242
Fecundation of plants ............ 393
Femur, fracture of neck of the .... 165
Ferguson, Dr. on contagion ...... 465
Fetid abscesses near mucous membranes ........................ 433
Fever, Dr. Stevens's crotchets on .. 323
Fingers, contraction of the........ 175
Fingers, fatal case of amputation of, 530
Fœtal monstrosity, curious case of, 162
Fracture, compound, of the skull, cases of ...................... 512
Fractured pelvis ................. 535
Fractured sternum ............... 536
Fractured scapula................ 537
Fractures of the spine ........... 14
Fungus of dura mater and cranium, 483

G.

Gonorrhœa in females............ 162
Graves, Dr. on various diseases .... 458
Green, Mr. on fracture of the cervix femoris ....................... 150

H.

Hæmaturia, Dr. Watson on ...... 491
Hæmorrhage after amputations .... 181
Hæmorrhage, secondary, Dr. Buchanan on ..................... 209
Hæmorrhage, uterine, Mr. Ingleby on ............................ 214
Hæmorrhage, uterine, Dr. Ramsbotham on ....................... 298
Hæmorrhage, uterine, Mr. Ingleby on .......................... 337
Hæmorrhage, in last months of pregnancy ....................... 347
Hæmorrhage, uterine, consequences of............................ 351
Hæmorrhage, from plurality of children ........................ 352
Hæmorrhage, internal and concealed 357
Hacket, Dr. his letter respecting Dr. Stevens ....................... 577
Hackman on softening of the spleen 257
Haunhemann, his doctrine homoiothique......................... 132
Hall, Dr. Marshall, on blood-letting, 248
Head, severe injury of the ........ 538
Heart, abscess of the ............ 170
Heart, disease of the............. 175
Heart, purulent cysts in the ...... 182
Heart, dilatation of the cavities of.. 183
Heart, disease of, unsuspected .... 406
Heart, disease of, remarks on .... 464
Heat, low power of resisting ...... 494
Hepatitis, ending in abscess ....... 182
Hernia, cases of, by Mr. Clement .. 45
Hernia, entero-vaginal, cases of.... 253
Hospital reports, Mr. Crampton on, 507
Hospital reports, further remarks on 510
Hospital reports, remarks on ...... 527
Hydrocele, diffused, of the cord.... 529
Hydrocephalus, cured by puncturing 187
Hydrophobia, injection into veins for 145
Hydro-rachitis, Mr. Stafford on .... 21
Hydrostatic bed, Dr. Arnott's...... 564

I.

Inanition, dissection after death from .......................... 185
Inflammation, Mr. James on ...... 411
Inflammation, theory of, considered 411
Inflammation from various poisons 419
Ingleby, Mr. on uterine hæmorrhage ......................... 214
Injection of encysted dropsy, death from .......................... 483
Injuries of the head, Mr. Crampton on ............................ 507
Iodine, use of, in scrofula ........ 169
Iodine for secondary eruptions .... 263
Iritis, cases of .................. 278
Iron, nitrate of, in diarrhœa ...... 214
Irritative fever from retained placenta ......................... 355

J

James, Mr. on Inflammation ...... 411
Jameson, Mr. his report on cholera 72
Jussieu's natural classification .... 398

K.

Karrait snake, bite from.......... 525
Keloide, M. Biett on ............ 382
King, Dr. on lithotrity and lithotomy 24

L.

Laceration of the urethra, cases of.. 533
Lactation, preternatural, cases of.. 201
Lee, Dr. on the placenta.......... 454
Lentigo, or freckles, M. Biett on .. 361
Lichen vulgaris, a diuretic ........ 523
Ligatures, flat, in aneurism ...... 444
Lithotomists, native, in India .... 526
Lithotomy in the perineum ...... 29
Lithotomy, cases of.............. 31
Lithotomy, hæmorrhage after .... 34
Lithotomy by the rectum ........ 35
Lithotomy above the pubes ...... 36
Lithotomy, case of, in a female child 233
Lithotomy for removal of piece of catheter.................... 528
Lithotrity and lithotomy, Dr. King on ......................... 24
Lithotrity ...................... 36
Lithotrity, instruments of ........ 37
Lithotrity, objections against, considered ..................... 38
Lithotrity, case of .............. 186
Liver, abscess of the ............ 206
Liver, cases of abscess of.......... 523
Lumbar abscess, cases of.......... 520
Lumbar abscess, remarks ........ 519
Lungs, case of emphysema of the .. 453
Lupus, M. Biett on .............. 363
Lupus, varieties of .............. 364
Lupus, diagnosis and treatment of.. 366
Lupus, Mr. Macfarlane on ........ 516
Lupus, cases of.................. 517
Lupus, causes of ................ 518

M.

M'Cormac, Dr. on palm oil ...... 207
Macfarlane, Mr. his clinical reports 515
Maculæ, M. Biett on ............ 360
Madras medical reports .......... 408
Malignant diseases all curable .... 328
Malingering .................... 262
Mammæ, neuralgia of the ........ 461
Man, natural history of .......... 170
Mandate, infamous, of Louis Philippe ........................ 445
Marshall, Mr. on the mortality of the metropolis ................ 243
Median nerve, partial excision of .. 489
Medical botany, outline of ........ 387
Medical constitution ............
Medulla, inflammation of ........ 179
Membranes, dropsy of the ........ 312
Menstruation in different countries 560
Midwifery, manual of, by Dr. Ryan 114
Midwifery, Dr. Ramsbotham on.... 289
Milk of cows before and after parturition .................... 485
Mortality of young recruits ...... 261

N.

Nævi materni, M. Biett on........ 362
Narcotics, externally applied...... 462
Neck, diffuse inflammation of the.. 417
Nervous system of plants ........ 391
Neuralgia of the mammæ ........ 461
Newburn, cholera at ............ 121
Newburn, situation of............ 122
Newburn, proportion of population attacked...................... 127
Nitras argenti for aphonia ........ 435
Nitric acid for toothache ........ 537

O.

Observations in Surgery and Pathology ........................ 43
Ocular illusions, Sir David Brewster on ........................ 467
Ocular illusions, instances of ...... 469
Ocular illusions, other instances of 478
Ophthalmia, catarrhal, cases of.... 267
Ophthalmia, purulent, cases of .... 269
Ophthalmia, strumous, cases of.... 273
Opium in uterine hæmorrhage .... 339
Optic nerves, anatomy and physiology of ...................... 447
Otto's pathological anatomy ...... 427

P.

Paganini, organization of ........ 177
Paralysis of portio dura, cured by moxa ...................... 437
Pathological anatomy of Otto ..... 427
Pelvis, fractured, case of......... 535
Pelvis fractured, fatal case of...... 539
Phlebitis, operation for .......... 543
Phlebitis, cases of................ 541
Phlegmasiæ, obscure forms of .... 442
Phosphorescence of the retina .... 470
Phthisis, Dr. Blackmore's notions on 402
Placenta, morbid conditions of the.. 344
Placenta, rupture of the.......... 349
Placenta, adhesion of the ........ 354
Placenta, Dr. Lee on the ........ 454
Polypus uteri, cases of, &c. ....... 317
Population, statistical researches on the .......................... 182
Poultry, epidemic disease among .. 439
Pregnancy, extra-uterine, probable cause of .................... 173
Pregnancy, extra-uterine, Dr. Ramsbotham on.................... 310

Pregnancy, doubtful, Dr. Ramsbotham on.................... 314
Propagation of plants ............ 394
Provincial medical and surgical association ..................... 574
Ptyalism, Dr. Graves on .......... 458
Puerperal fever, Dr. Ryan on...... 115
Puerperal fever, symptoms of...... 116
Puerperal fever, treatment of...... 118
Puerperal convulsions, Dr. Ramsbotham on.................... 304
Purpura, M. Biett on ............ 379
Pus discharged from ear, after injury of head .................... 538
Pylorus, disease of, cured by narcotics ......................... 168

Q.

Quinine, report on the use of...... 409
Quinine, affections of head produced by ........................ 522

R.

Rainey, Mr. on arsenic ........... 198
Ramollissement of spinal marrow.. 23
Ramsbotham, Dr. on Midwifery.... 289
Ratiocination of Dr. Stevens ...... 323
Recto-vaginal fistula, cured ...... 486
Rectum, lithotomy by............. 35
Reid's medical botany...... ..... 387
Reports on the cholera in Hindostan 57
Reports of the Madras Board...... 408
Respiration, experiments on ...... 190
Retention of urine, Dr. Ducamp on, 49
Retroversio uteri, remarks on .... 315
Revulsive bleedings, advantages of 188
Rheumatism, treated by morph. acet. 436
Rhino-plastic operation .......... 172
Roots of plants, functions of the .. 392
Roseola, caused by cubebs and copaiba ......................... 185
Ryan, Dr. on midwifery .......... 114

S.

Saline treatment of Dr. Stevens.... 331
Saline treatment, results of ...... 333
Saline treatment, analysis of those results ...................... 334
Salines, powers of, in fever........ 329
Scarlet fever, hints on the ........ 176
Schloss, Mr. his anatomical models 564
Sciatica, cured by bleeding........ 436
Scrofula, treatment of, by iodine .. 169
Scrofula of bones, cases of ........ 439
Scurvy, Stevens' discoveries in .... 330
Sebaceous follicles, disease of the .. 381
Secret remedies ................ 161
Shoulder-presentations, Dr. Ramsbotham on.................... 292
Shoulder-presentations, cases of .. 294
Skin, Biett on diseases of the ...... 360
Soldiers, Austrian, strictness in discharge of ...................... 260
Somerville, Dr. his circulars on the Anatomy Act................. 570
Spectral illusions, Sir D. Brewster on ......................... 473
Spectral illusions, his theory of.... 474
Spectral illusions, remarks on the theory ...................... 477
Spina bifida, Mr. Stafford on ...... 2
Spina bifida, cases of ............ 4
Spina bifida, treatment of ........ 5
Spine, injuries of the ............ 8
Spine, fractures of the............ 14
Spine, dislocations of the ......... 15
Spine, cancellous structure of .... 17
Spine, ulceration, &c. of.......... 19
Spleen, softening of, Hackman on.. 257
Stafford, Mr. on injuries of the spine 1
Sternum, case of fracture of ...... 536
Stevens, Dr. on the blood ........ 321
Stevens, Dr. his modesty.......... 327
Stone, Dr. King on treatment of .. 24
Strangulated hernia, case of ..... 156
Strength, remarkable feats of .... 481
Stricture, treatment of by caustic.. 50
Stricture, instruments used in .... 51
Sweating fever, epidemic in France 446
Syphilitic eruptions, M. Biett on .. 369
Syphilitic eruptions, exanthematous 370
Syphilitic eruptions, vesicular .... 370
Syphilitic eruptions, pustular...... 371
Syphilitic eruptions, tubercular.... 372
Syphilitic eruptions, papular ...... 373
Syphilitic eruptions, scaly ........ 373
Syphilitic eruptions, diagnosis of .. 374
Syphilitic eruptions, treatment of.. 376

T.

Thymus gland, anatomy of the .... 139
Thymus gland in the human subject 140
Thymus gland, physiology of the .. 142
Thymus gland, disease of ........ 144
Tic douloureux, case of .......... 196
Tic douloureux of a stump, case of 489
Tod, Mr. on the ear ............. 431
Tumor, the remains of a hernial sac 185
Twin-labours, treatment of ...... 307
Typhus, French treatment of...... 444

U.

Undercliff, Isle of Wight.......... 573
Urethra, Dr. Ducamp on stricture of 49
Urethra, lacerated, fatal cases of .. 533
Uric acid, Mr. Rodweiss on ...... 486
Urine in diseases of the spine...... 10
Urine, difficulty of passing after gonorrhœa ..................... 44
Urine, retention of ............. 178

Uterine hæmorrhage, Dr. Ramsbotham on.................... 299
Uterine hæmorrhage, cases of .... 301
Uterine hæmorrhage, Mr. Ingleby on 337
Uterine hæmorrhage, internal .... 349
Uterus, singular tumor from the .. 152
Uterus, retroversion of .......... 313
Uterus, polypus of ............... 315
Uterus, rupture of ............... 318
Uterus, apoplexy of............... 319
Uterus, action of the ergot on the.. 341
Uterus, spasmodic contraction of .. 353

V.

Vagina, abscesses opening into the.. 222
Vagina, cases of contraction of the.. 224
Vaginal plug, Mr. Ingleby on the .. 345
Varicose tumour ................. 184
Vascular system, Dr. Wise on diseases of the ................. 221
Velpeau, M. on fetid abscesses .... 433
Vessels, state of, in inflammation .. 411
Vesico-vaginal fistula cured ...... 486
Vitreous humour regenerated...... 486
Volcano of pestilence under London 326
Vomiting in pregnancy .......... 337

W.

Watson, Dr. on hæmaturia........ 491
Webster, Dr. on cholera .......... 383
Westminster Ophthalmic Hospital.. 265
Westminster Ophthalmic Hospital, half-yearly report from ........ 284

INDIGESTION—CHANGE OF AIR.

AN ESSAY on INDIGESTION, as the Source of various Maladies, mental and corporeal, with an improved Method of Treatment, medicinal and dietetic.

By JAMES JOHNSON, M.D. Physician Extraordinary to the King.

*Seventh Edition, price* 6*s*. 6*d*.

ALSO, BY THE SAME AUTHOR.

CHANGE of AIR, or the PURSUIT of HEALTH and RECREATION; illustrating the beneficial Influence of Bodily Exercise, Change of Scene, and Temporary Relaxation from Business, as restorative and preservative of Health.

*Third Edition (greatly improved) price* 8*s*. 6*d*.

S. Highley, 32, Fleet-street.

CRITICAL NOTICES.

" Of all the popular Tours, of which British literature has recently been so prolific, this is immeasurably the best. To attempt an analysis of a work embracing such a treasure of anecdote and instruction, would be an idle task. There is no class of general readers which may not derive pleasure and profit from the perusal of this volume."—BALLOT.

" Dr. JOHNSON is a vigorous and independent thinker, while his opinions are slightly tinctured with cynicism, which gives them an agreeable relish. His style is clear, bold, and expressive; so that when he least aims at effect, he leaves a more vivid impression on us of the object of his reflections, than others would by an elaborate description."—MORNING HERALD.

" The author, out of his abundant stock of knowledge and reflection, has constructed a volume with which all classes will be pleased."—ATLAS.

" The medical portion embraces numerous remarks which we would recommend all invalids and their medical advisers to peruse, before they decide upon the dangerous experiment of foreign travel."—SPECTATOR.

" In his descriptions of the different countries he passed through, and the many objects of curiosity that attracted his notice, Dr. JOHNSON has preserved a freshness and originality, that reflect high credit upon his talent, as a writer and acute observer."—LONDON MED & PHYS. JOURN.

" The present publication is the most entertaining and edifying that has issued from the press for many years."—GAZETTE OF HEALTH.

" This work is so spirited, so full of sound moral reflection—so correct, and so impartial, that we scarcely know where to find its equal. It is a classical and philosophical tour. It is impossible to dip into any part of it, without having the attention rivetted and the fancy pleased."—LONDON MED. AND SURG. JOURNAL.

